# Regulation of Macronutrients in Insulin Resistance and Glucose Homeostasis during Type 2 Diabetes Mellitus

**DOI:** 10.3390/nu15214671

**Published:** 2023-11-04

**Authors:** Wanbao Yang, Wen Jiang, Shaodong Guo

**Affiliations:** Department of Nutrition, College of Agriculture and Life Sciences, Texas A&M University, College Station, TX 77843, USA; wanbao.yang@ag.tamu.edu (W.Y.); jiangwen@tamu.edu (W.J.)

**Keywords:** macronutrients, insulin resistance, glucose homeostasis, type 2 diabetes mellitus

## Abstract

Insulin resistance is an important feature of metabolic syndrome and a precursor of type 2 diabetes mellitus (T2DM). Overnutrition-induced obesity is a major risk factor for the development of insulin resistance and T2DM. The intake of macronutrients plays a key role in maintaining energy balance. The components of macronutrients distinctly regulate insulin sensitivity and glucose homeostasis. Precisely adjusting the beneficial food compound intake is important for the prevention of insulin resistance and T2DM. Here, we reviewed the effects of different components of macronutrients on insulin sensitivity and their underlying mechanisms, including fructose, dietary fiber, saturated and unsaturated fatty acids, and amino acids. Understanding the diet-gene interaction will help us to better uncover the molecular mechanisms of T2DM and promote the application of precision nutrition in practice by integrating multi-omics analysis.

## 1. Introduction

Type 2 diabetes mellitus (T2DM) has become a global health issue that is tightly correlated with the prevalence of obesity and other chronic diseases. T2DM is caused by systemic insulin resistance and impaired insulin secretion in pancreatic β-cells, leading to disorders of carbohydrate, protein, and lipid metabolism [1]. Insulin resistance is a hallmark of prediabetes and gradually contributes to the development of T2DM. Insulin resistance refers to an impaired ability of insulin to lower blood glucose in target tissues at a normal plasma insulin level. Pancreatic β-cells secrete excessive insulin to compensate for the outcome of insulin resistance. Thus, fasting plasma insulin levels rise, and hyperinsulinemia eventually develops [2]. Although it is controversial about the primary defect between insulin resistance and hyperinsulinemia, they together culminate in eventual β-cell failure, resulting in hyperglycemia [3,4].

Insulin receptor (IR) is a tyrosine kinase receptor that is activated upon insulin binding, recruiting downstream substrates such as insulin receptor substrate (IRS). This initiation sets off the proximal insulin signaling pathway. Phosphorylated IRS, in turn, triggers the phosphoinositide 3-kinase (PI3K) → protein kinase B (AKT) signaling cascade, regulating the activity of critical distal downstream targets, including glucose transporter type 4 (GLUT4), mammalian target of rapamycin complex 1 (mTORC1), and forkhead box protein O1 (FoxO1), regulating glucose and energy homeostasis [5,6]. Insulin resistance can manifest at multiple cellular levels, including the desensitization of the insulin receptor at the cell surface, inhibition of IRS function via protein degradation, suppression of PI3K activity, an inability to inhibit FoxO1-induced transcriptional changes, and reduced insulin clearance from the bloodstream [6,7]. Additionally, IR → Carcinoembryonic antigen-related cell adhesion molecule 1 (CEACAM1) signaling pathway-mediated hepatic insulin clearance is pivotal for the regulation of insulin homeostasis [7,8]. Impaired insulin clearance leads to progressive insulin resistance, potentially due to the development of chronic hyperinsulinemia.

Metabolic organs cooperatively respond to nutrient intake and maintain energy balance. The interaction of nutrients, especially macronutrients, with the gastrointestinal tract stimulates the release of incretin hormones such as glucose-dependent insulinotropic polypeptide (GIP) and glucagon-like peptide-1 (GLP-1) [9]. GLP-1 improves insulin sensitivity in peripheral tissues through increasing insulin secretion, attenuating inflammation response and endoplasmic reticulum (ER) stress, increasing GLUT-4 expression, and enhancing insulin signal transduction [10,11,12,13]. GIP stimulates the release of both insulin and glucagon in the pancreas. Therefore, incretin hormones play a key role in regulating insulin sensitivity and glucose homeostasis. Nutrient ingestion, especially carbohydrates, increases blood glucose and stimulates insulin secretion in the pancreas, thereby suppressing glucose production in the liver, increasing lipogenesis in the adipose tissue, promoting glucose uptake in the skeletal muscle, and regulating glucose homeostasis. Overnutrition-induced obesity is highly related to insulin resistance, primarily through multiple pathological mechanisms. The nutrient-induced GLP-1 secretion is significantly reduced in obese individuals [14,15], which may contribute to systemic insulin resistance. During overnutrition, toxic metabolites including ceramides, diacylglycerol (DAG), and nonesterified fatty acids (NEFA) accumulate and stimulate the activity of protein kinase C (PKC), leading to the Ser/Thr phosphorylation of IR and IRS, thereby impairing insulin sensitivity [2]. Overnutrition elevates branched-chain amino acid levels in the bloodstream, activating mTORC1 and inhibiting IRS function [16]. Proinflammatory cytokine levels in circulation are increased during obesity, which induces Ser/Thr phosphorylation of IRS and inhibits insulin signaling by activating JNK and IKKβ [17,18,19]. Hyperinsulinemia associated with obesity leads to insulin resistance by inhibiting the activity of IRS [20]. In this review, we will focus on the effect of macronutrients on insulin resistance and the underlying mechanisms.

## 2. Insulin and Glucagon Action and Insulin Resistance

### 2.1. Molecular Basis of Insulin and Glucagon Signaling

Insulin and glucagon are pivotal hormones that play essential roles in regulating glucose homeostasis. These two hormones cooperate tightly to maintain blood glucose levels within a narrow range, preventing both hyperglycemia and hypoglycemia under certain health conditions. The glucagon-to-insulin ratio is strongly linked to hyperglycemia in patients with type 2 diabetes [21].

Insulin, a peptide hormone secreted from pancreatic β-cells, plays a critical role in orchestrating the anabolic response to nutrient intake, especially the intake of glucose, fatty acids, and amino acids [22]. Insulin regulates blood glucose balance by increasing glucose uptake in skeletal muscle and fat tissues while suppressing hepatic glucose production (HGP). Insulin actions are induced by the intrinsic tyrosine kinase activity of the insulin receptor (IR). Insulin triggers conformational changes and autophosphorylation of IR, leading to the recruitment and phosphorylation of IRS and SH3-containing protein (Shc). IRS activates the PI3K → AKT pathway to govern insulin’s metabolic functions, while Shc activates the Ras → MAPK pathway, which mediates growth and differentiation at cellular and organismal levels [23]. Additionally, IR also exerts control over apoptosis, senescence, and the cell cycle, independent of ligand and tyrosine kinase activity [24]. In skeletal muscle and adipose tissues, insulin promotes the translocation of GLUT4 from the cytoplasm to the cell membrane, thereby promoting glucose uptake [25]. Insulin-stimulated GLUT4 trafficking is mediated by the PI3K → AKT → AS160 signaling cascade [26,27,28]. AS160, a Rab GTPase-activating protein, maintains Rab proteins in an inactive GDP form and results in GLUT4 retention in the cytoplasm. Phosphorylation of AS160 by AKT inactivates its Rab GTPase activity, resulting in an increase in the active GTP form of Rab, thereby promoting GLUT4 trafficking [25,26]. In addition, AKT-induced PIKfyve phosphorylation plays a role in regulating insulin-stimulated GLUT4 trafficking, potentially through PtdIns 3,5-P2 [29,30,31]. In the liver, insulin inhibits glycogenolysis and gluconeogenesis, thus suppressing glucose release. Insulin activates glycogen synthase by the PI3K → AKT → GSK-3 signaling pathway. AKT stimulates phosphorylation of GSK-3 and inhibits its kinase activity, thereby dephosphorylating glycogen synthase, stimulating its activity, and promoting glycogen accumulation [32]. The IRS → PI3K → AKT → FoxO1 signaling pathway in the liver is primarily responsible for insulin action on glucose homeostasis [33]. Insulin stimulates phosphorylation of FoxO1 at S253 via the activation of AKT, thereby decreasing FoxO1-induced expression of genes responsible for gluconeogenesis such as *phosphoenolpyruvate carboxykinase 1* (*Pck1*) and *glucose-6-phosphatase* (*G6pase*) and suppressing HGP [34,35,36]. The central nervous system (CNS), such as the hypothalamus, plays a pivotal role in regulating systemic insulin sensitivity and glucose homeostasis [37]. The activation of insulin signaling in agouti-related peptide (AgRP) but not pro-opiomelanocortin (POMC) neurons suppresses HGP through the hepatic vagal nerve [38]. Insulin induces the hyperpolarization of AgRP neurons and decreases their activity, thus leading to the activation of IL-6-STAT3 signaling and downregulating hepatic gluconeogenic genes, including *Pck1* and *G6pase* [38,39]. Additionally, hypothalamic insulin activation inhibits lipolysis and promotes lipogenesis in white adipose tissue, which may contribute to the central regulation of HGP by limiting the supply of glycerol substrate for gluconeogenesis [40]. In humans, acute intranasal insulin delivery increases systemic insulin sensitivity during hyperinsulinemic-euglycemic clamp and enhances postprandial thermogenesis [41,42]. Chronically daily intranasal insulin administration for 8 weeks decreases body weight and adiposity in healthy men [43]. These results indicate that CNS insulin signaling regulates systemic insulin sensitivity and glucose homeostasis in both humans and mice.

Glucagon is secreted by the pancreatic α-cells, acting as a catabolic hormone and regulating glucose homeostasis. Glucagon promotes HGP through stimulation of glycogenolysis and gluconeogenesis, thereby maintaining euglycemia under fasting conditions. The action of glucagon is mediated by the glucagon receptor (Gcgr), a G protein-coupled receptor [44]. Upon glucagon binding, Gcgr activates Gs protein, thereby stimulating adenylate cyclase, elevating cyclic adenosine monophosphorylate (cAMP), and then activating cAMP-dependent protein kinase A (PKA) and exchange protein directly activated by cAMP 2 (EPAC2) [45]. Active PKA, in turn, stimulates the activity of phosphorylase kinase, which then converts glycogen phosphorylase b (PYG b) into the active form PYG a and promotes glycogen breakdown [46]. Glucagon promotes gluconeogenesis through the activation of two key transcriptional factors, cAMP response element-binding protein (CREB) and FoxO1 [47]. Activation of PKA by glucagon induces phosphorylation of CREB at S133, resulting in the formation of the CREB-TORC2 complex and increasing transcription of its downstream gluconeogenic genes, including *Ppargc1a*, *Pck1*, and *G6pase* [48,49]. Glucagon stimulates phosphorylation of FoxO1 at S273 via both PKA and EPAC2 → p38 signaling pathways, enhancing FoxO1 stability and nuclear localization [50,51]. Additionally, glucagon stimulates amino acid uptake and subsequent catabolism, increasing the substrate availability of amino acid metabolites and promoting gluconeogenesis [52]. Hepatic acetyl-CoA allosterically stimulates the activity of pyruvate carboxylase and increases gluconeogenesis in the liver [53]. Glucagon stimulates intrahepatic lipolysis, induces fatty acid oxidation, and increases acetyl-CoA levels, thereby promoting hepatic gluconeogenesis [54]. However, the mediobasal hypothalamus glucagon infusion inhibits HGP through glucagon receptor → PKA signaling [55]. This hypothalamic glucagon action on HGP is mediated by K_ATP_ channel-dependent mechanisms [56]. These results indicate that there exists a self-regulatory feedback loop to fine-tune glucagon-induced HGP.

### 2.2. The Molecular Basis of Insulin Resistance by Targeting FoxO1

**Figure 1:**
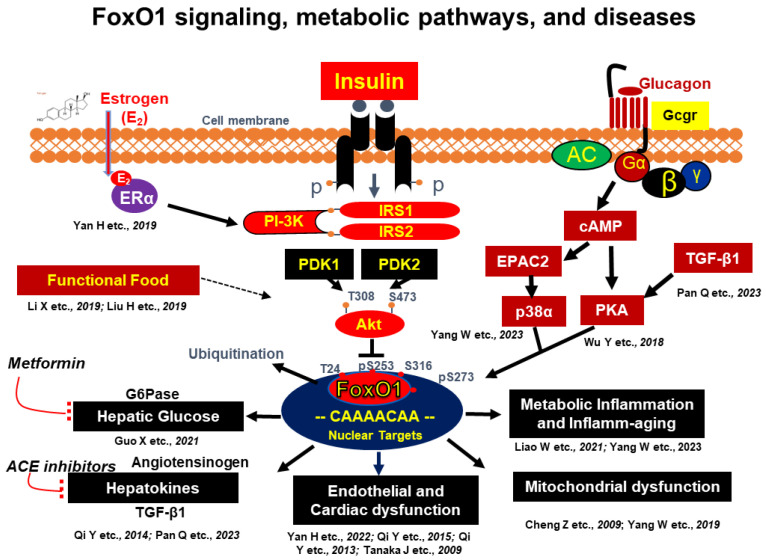
FoxO1 plays a pivotal role as a metabolic regulator in hormonal regulation, metformin function, and the influence of bioactive functional foods in both health and disease. Insulin and estrogen stimulate FoxO1 phosphorylation at T24, S253, and S316 through PI3K → AKT signaling, thereby inhibiting FoxO1 activity. Glucagon stimulates FoxO1 phosphorylation at S273 through cAMP → PKA and cAMP → EPAC2 → p38 signaling, thereby increasing FoxO1 activity. Activation of FoxO1 promotes hepatic glucose production, increases hepatokine secretion, impairs cardiac and hepatic mitochondrial function, and induces inflammation during metabolic stress and aging. AC, adenylyl cyclase; AKT, protein kinase B; cAMP, cyclic adenosine monophosphate; EPAC2, exchange protein directly activated by cAMP 2; ERα, estrogen receptor α; FoxO1, forkhead/winged helix transcription factor O-class member 1; Gcgr, glucagon receptor; G6Pase, glucose-6-phosphatase; IRS, insulin receptor substrate; P, phosphorylation; PDK, phosphoinositide-dependent protein kinase; PKA, protein kinase A; PI3K, phosphatidylinositol 3-kinase. →: Activation; 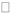: Inhibition [50,51,61,62,63,64,65,66,67,68,69,70,73,76,77].

The molecular mechanisms of insulin resistance have been extensively reviewed [2,5,6,23]. During obesity, lipotoxicity, chronic inflammation, hyperglycemia, hyperinsulinemia, mitochondrial dysfunction, and ER stress stimulate the activity of Ser/Thr kinase and impair insulin sensitivity by phosphorylating IR, IRS, and AKT proteins, resulting in insulin resistance. Insulin resistance is a critical mechanism underlying various metabolic disorders. Over the past decade, we have proven that FoxO1 is one of the key factors that link insulin resistance and metabolic disorders. Studies from many labs, including our own, have demonstrated that FoxO1 promotes HGP by upregulating the expression of gluconeogenic genes, including *Pck1* and *G6pase* [57,58]. Insulin stimulates the activity of AKT and phosphorylates FoxO1 at T24, S253, and S316, thereby suppressing FoxO1 activity and inhibiting HGP [35,36,59,60]. Deletion of liver IRS1 and 2 in mice induces systemic insulin resistance, leading to diabetic symptoms that include hyperglycemia and hyperinsulinemia. Notably, these diabetic symptoms are normalized when hepatic FoxO1 is deficient [33]. We recently uncovered that glucagon stimulates the phosphorylation of FoxO1-S273 through the cAMP → PKA and cAMP → EPAC2 → p38α signaling pathways; this enhances FoxO1 protein stability and promotes its nuclear localization. We also found that FoxO1-S273 phosphorylation prevents insulin-mediated FoxO1 degradation in vitro and impairs glucose tolerance in vivo [50,51]. These findings suggest that FoxO1 is a pivotal mediator connecting glucagon and insulin signaling in the regulation of glucose homeostasis. In addition, FoxO1 plays a crucial role in aging-induced glucose dysregulation and chronic inflammation. We found that the activity of FoxO1 was increased in the livers of old mice. Inhibition of FoxO1 significantly improves glucose homeostasis and attenuates chronic inflammation in Kupffer cells during aging [61]. Activation of FoxO1 impairs hepatic mitochondrial function by controlling heme homeostasis, thereby contributing to insulin resistance-mediated hepatic mitochondrial dysfunction [62,63]. Heme oxygenase-1 (HO-1) is one of the target genes regulated by FoxO1 [62]. HO-1 gain-of-function increases ferrous iron levels and promotes inflammation in livers [64]; these results indicate that the FoxO1 → HO-1 signaling pathway plays a key role in ferrous iron overload and chronic inflammation under insulin-resistant conditions. Activation of FoxO1 increases TGF-β1 levels, impairing glucose and energy metabolism as well as exacerbating CCL4-induced liver fibrosis [65,66]. In male mice, heart insulin resistance caused by cardiac IRS1 and IRS2 ablation induces heart failure and gradually leads to death, which is rescued by cardiac FoxO1 deletion [20,67]. Cardiac FoxO1 deficiency also improves heart function in both db/db and high-fat diet-induced obese male mice [67]. Additionally, we found that FoxO1 modulates blood pressure by regulating expression levels of the angiotensinogen gene in mice [68]. In addition, we have provided evidence that FoxO1 mediates the beneficial effect of estrogen on glucose dysregulation and heart failure under insulin resistance [69,70]; these results indicate that FoxO1 signaling contributes to the sex dimorphism observed in heart diseases and diabetes. Endothelial dysfunction is highly associated with several diabetes-related vascular complications [71,72]. In diabetic conditions, hyperglycemia, excessive free fatty acids, and insulin resistance induce endothelial dysfunction through increased inflammation and reactive oxygen species (ROS) as well as decreased NO bioavailability, thereby leading to cardiovascular diseases [71]. FoxO1 gain-of-function induces lipid peroxidation and eNOS dysfunction in vascular endothelial cells, and FoxO1 loss-of-function blocks hyperglycemia-induced dysfunction of endothelial cells [73]. These results indicate that FoxO1 is a key mediator that links hyperglycemia to endothelial dysfunction. In summary, FoxO1 plays a pivotal role in insulin resistance-induced hyperglycemia, chronic inflammation, hepatic mitochondrial dysfunction, liver fibrosis, heart failure, and hypertension. Thus, targeting FoxO1 is a promising strategy for the treatment of insulin resistance-associated metabolic diseases. Metformin is a well-established drug for the treatment of T2DM [74]. Epigallocatechin gallate (EGCG), the most abundant catechin in green tea, protects against the development of T2DM [75]. Indeed, our studies have shown that metformin and EGCG inhibit FoxO1-S273 phosphorylation, thus suppressing FoxO1 activity and improving glucose homeostasis [76,77] (Figure 1).

## 3. Dietary Carbohydrates and Glucose Homeostasis

Dietary carbohydrates encompass monosaccharides including glucose, fructose, and galactose; disaccharides including sucrose, lactose, and maltose; oligosaccharides typically consisting of 3–9 molecules in chain length; polysaccharides, notably starch; and dietary fiber. The sugars normally contain mono- and disaccharides. Dietary sugars and starches mainly contribute to energy intake [78]. Carbohydrate intake stimulates insulin secretion, which increases fat storage and decreases lipolysis and fatty acid oxidation. A low-carbohydrate diet improves metabolic syndrome, including lipid and glucose homeostasis [79].

### 3.1. Dietary Sugars and Insulin Resistance

The increased consumption of refined and simple carbohydrates promotes the development of insulin resistance and T2DM. The World Health Organization and the Food and Agriculture Organization recommended a restriction on free sugar intake to prevent T2DM and obesity [80]. The clinical studies show that high fructose intake (>250 g/day) decreases insulin sensitivity and increases adiposity in both healthy and obese individuals [81,82]. However, moderate fructose intake (<100 g/day) has a limited effect on insulin sensitivity and metabolic health [83]. The overconsumption of dietary sugars promotes the development of insulin resistance, both directly and indirectly. Dietary sugar overconsumption promotes a positive energy balance, thereby increasing body weight and fat deposition and indirectly leading to insulin resistance and glucose dysregulation. Additionally, fructose reduces hypothalamic malonyl-CoA levels and increases the hunger hormone ghrelin [84,85,86], thereby stimulating appetite, increasing body weight, and impairing insulin sensitivity. On the other hand, fructose, the sweetest of all naturally occurring carbohydrates, directly causes insulin resistance via increasing hepatic lipid accumulation and promoting inflammation. Fructose is a highly lipogenic sugar and is mainly metabolized in the liver, with very little entering the systemic circulation [87]. Fructose is transported into the liver by GLUT2, phosphorylated into fructose-1-phosphate, and further metabolized to dihydroxyacetone phosphate and glyceraldehyde 3-phosphate, which stimulates de novo lipogenesis [88]. Administration of fructose to mice significantly increases the activity of carbohydrate-responsive element-binding protein (ChREBP) and sterol regulatory element-binding protein 1 (SREBP-1c), two important transcription factors stimulating lipogenesis [89,90]. Additionally, fructose intake has been shown to block hepatic fatty acid oxidation, potentially via uric acid-induced mitochondrial oxidative stress [91,92]. Increased hepatic lipid accumulation induces hepatic insulin resistance through PKC-mediated phosphorylation of the insulin receptor at Thr 1160, thereby impairing glucose homeostasis and promoting the development of T2DM [93]. The human studies from Havel’s group showed that fructose- but not glucose-sweetened beverage consumption led to a significant increase in lipogenesis, dyslipidemia, and circulating uric acid levels, and a significant decrease in fatty acid oxidation and insulin sensitivity [81]. These different effects between fructose and glucose may be attributed to the marked distinctions in their hepatic metabolism, such as their entry into the glycolytic pathway and the fate of their intermediate metabolites [88].

On the other hand, dietary sugar or fructose impairs immune homeostasis and promotes inflammation, thereby leading to insulin resistance. Dietary fructose is transported into the enterocyte through a specific fructose transporter, GLUT5. Healthy individuals can absorb up to 25 g of fructose [94]. High-fructose diet-induced fructose overload leads to intestinal barrier deterioration and alters gut microbiota composition, thereby increasing endotoxin release, promoting systemic inflammation, and leading to insulin resistance [95,96]. The impairment of the intestinal tissue repair and the gut microbiota environment by fructose leads to intestinal barrier deterioration [97]. Fructose-stimulated endotoxin release increases TNF production in liver macrophages, mediating fructose-induced hepatic insulin resistance and lipogenesis [97,98]. Dietary sugar regulates the crosstalk between the microbiota and intestinal immunity to control insulin resistance and other metabolic disorders. Dietary sugar is a key to disrupting intestinal immune homeostasis via eliminating Th17-inducing microbiota (especially segmented filamentous bacteria). Dietary sugar increases a member of Erysipelotrichaceae to eliminate Th17-inducing microbiota, decreasing Th17 cells, increasing intestinal lipid absorption, and promoting insulin resistance [99]. Of note, a rodent study showed that high fructose intake (30% *w*/*v*) leads to a more pronounced glucose intolerance in males than in females [100]. In humans, sex differences in the effect of fructose on uric acid concentration but not glucose homeostasis are observed [101]. However, a larger sample size is warranted to investigate the sex difference in fructose-impaired glucose homeostasis in humans.

Low-calorie sweeteners (LCS), including aspartame, saccharin, sucralose, and steviol glycoside, provide an alternative to added sugars and have been used to control body weight gain [102]. The effects of LCS on reduction of caloric intake and body weight gain make LCS a promising approach to controlling blood glucose in patients with T2DM. However, the beneficial effects of LCS are controversial in human studies. In some randomized controlled trials (RCTs), LCSs significantly decreased body weight, body mass index, fat mass, and waist circumference [103]. Contrarily, results from some RCTs fail to show a significant effect of LCSs on weight management [104]. Several observational studies provide evidence that consumption of LCSs is associated with a significantly increased risk of T2DM and a higher incidence of abdominal obesity [105,106,107]. Further studies are warranted to confirm the effects of LCSs on metabolic diseases.

### 3.2. Dietary Fibers and Insulin Resistance

Dietary fiber is composed of highly complicated substances that include any indigestible carbohydrate and lignin that cannot be degraded in the upper gastrointestinal tract. The viscous, gel-forming, and soluble dietary fiber derived from fruit and certain vegetables suppresses the absorption of macronutrients, reduces postprandial glucose response, and improves lipid profiles. A meta-analysis with 176,117 subjects showed that higher insoluble cereal fiber intake was significantly associated with a lower incidence of diabetes, whereas fruit and vegetable fiber intake had no significant association with the risk of T2DM [108]. Consistently, the consumption of insoluble dietary fiber significantly increases insulin sensitivity in both healthy and diabetic individuals [109,110,111]. However, other studies provide evidence that soluble dietary fibers decrease postprandial blood glucose and increase insulin sensitivity in both nondiabetic and diabetic subjects [112,113]. Compared to soluble dietary fiber, insoluble dietary fiber has a better effect on attenuation of high-fat diet-induced obesity, body fat composition, and insulin resistance in mice [114]. Short-chain fatty acids (SCFAs), including butyrate, propionate, and acetate, are generated by the gut microbiota through fermenting non-digestible dietary fibers and play an important role in the beneficial effects of dietary fibers on insulin resistance and glucose homeostasis. SCFAs stimulate the secretion of the gut hormone anorexigenic peptide YY (PYY) through free fatty acid receptor 2 (FFAR2), thereby increasing satiety, decreasing body weight, and improving insulin sensitivity [115]. Additionally, SCFAs acetate and propionate increase the secretion of GLP-1 via FFAR2 and 3, contributing to the improvement in glucose tolerance and hepatic insulin sensitivity [116,117]. Wu et al. group reported that glucose infusion in the distal small intestine largely increased plasma GLP-1, with a minimal increase after proximal small intestine glucose infusion [118]. These results suggest that the distal small intestine plays a key role in regulating GLP-1 secretion and modulating glucose homeostasis. Dietary fiber reduces the rate of carbohydrate absorption and increases the interaction between the distal gut and carbohydrate, thereby promoting GLP-1 secretion and improving insulin sensitivity [119,120]. Previous studies have shown that pro-inflammatory markers are reduced by high dietary fiber consumption in both human and rodent models. A randomized trial with 120 obese women showed that consumption of a high-dietary-fiber diet decreased serum proinflammatory marker levels, including IL-6, IL-18, and C-reactive protein (CRP) [121]. In the obese rat model, plasma TNF levels were significantly reduced by the supplementation of dietary fibers for 25 weeks [122]. The anti-inflammatory effect of dietary fiber is potentially attributed to the product of fiber fermentation, butyrate. Previous studies indicate that butyrate attenuates pro-inflammation by inhibiting NF-κB signaling, decreasing IL-12, and increasing IL-10 production in human immune cells [123,124]. Thus, dietary fiber attenuates pro-inflammatory activity through SCFA butyrate, thereby increasing insulin sensitivity and improving glucose tolerance. Intestinal gluconeogenesis-derived glucose is sensed by the gastrointestinal nervous system around the portal vein, transducing signals to the brain regions and regulating energy homeostasis [125,126]. SCFAs, including butyrate and propionate, contribute to intestinal gluconeogenesis and transmit signals to brain regions, thereby executing beneficial effects on insulin sensitivity and glucose metabolism [127].

## 4. Lipid Metabolism and Glucose Homeostasis

### 4.1. Dietary Fat and Insulin Resistance

Dietary fat is highly associated with the incidence of insulin resistance in both mice and humans. In mice, high-fat diet (60% calories derive from fat) feeding for 13 weeks leads to insulin resistance and glucose intolerance [76]. In humans, a higher total fat intake is significantly associated with increased fasting insulin levels, HbA1c, and 2-h post-load glucose levels [128,129,130]. It is estimated that an increase in dietary fat intake of 40 g/d is correlated with a 3.4-fold increased risk of T2DM [131]. However, several population studies failed to show a significant correlation between total fat intake and the incidence of T2DM, which may be attributed to the quantity and quality of dietary fat. There are mainly two kinds of dietary fats: saturated and unsaturated fats, which play different roles in the development of insulin resistance. Most epidemiologic and clinical studies consistently show that saturated fat intake is tightly associated with the pathogenesis of insulin resistance and T2DM, whereas polyunsaturated fat intake is significantly associated with improved insulin sensitivity and glucose tolerance [129,130,132,133,134]. Of note, the beneficial effect of a monounsaturated fatty acid diet is attenuated when total fat intake is over 38% [135]. Additionally, fatty acids with different lengths of carbon chains have distinct effects on insulin sensitivity. Long-chain fatty acids (LCFAs, >C16) are the major fatty acids in the western diet and are highly associated with insulin resistance and impaired glucose homeostasis [136,137]. Medium-chain fatty acids (MCFAs, C8–C12) lead to a decrease in adiposity and an improvement in insulin action compared to LCFAs with equal calories, which is potentially attributed to increased energy expenditure and fatty acid oxidation by MCFAs [138,139,140]. Short-chain fatty acids (SCFAs, C2–C6) are produced from the fermentation of indigestible food by the gut microbiome. SCFAs exert a beneficial effect on insulin sensitivity by targeting adipose tissue, skeletal muscle, and liver [141,142]. Thus, the quality and quantity of dietary fat intake are pivotal in the development of insulin resistance and T2DM.

### 4.2. Molecular Mechanisms of FFA-Induced Insulin Resistance

Lipid overload induces insulin resistance in skeletal muscle and liver, thereby leading to defects in insulin-mediated glucose uptake and suppression of HGP, respectively. The detrimental effect of lipid overload on insulin sensitivity is mainly attributed to the increased circulating free fatty acid (FFA) levels, especially saturated free fatty acids (SFAs) [143]. In the KANWU study, high SFA diet intervention significantly impairs insulin sensitivity in both healthy men and women [144]. Aberrant SFA levels stimulate the inflammatory pathway, increase ROS production, generate insulin resistance-associated lipid production, and impair protein folding homeostasis, thereby leading to insulin resistance. Several previous studies showed that SFA treatment activated pro-inflammatory signaling in adipocytes, hepatocytes, and macrophages [145,146,147], suggesting that SFA is a pro-inflammatory lipid compound. The pro-inflammatory role of SFAs is partially mediated by activation of TLR2/4 signaling [146,148,149,150,151]. It is proposed that SFAs activate TLR2/4 signaling indirectly through binding to TLR coreceptors (such as CD36), promoting dimerization of TLR4, and inducing redistribution of c-Src in lipid rafts [152,153,154,155]. Activation of SFA → TLR signaling increases inflammatory cytokines such as TNF and activates JNK, thereby inhibiting phosphorylation of IRS1 and attenuating insulin sensitivity [156,157]. In addition, SFAs also induce the activation of the NLRP3-APC inflammasome through increasing mitochondrial ROS and impairing autophagy, thereby promoting IL-1β release and leading to insulin resistance [158]. DAGs, the lipid products of SFAs, are significantly linked to lipid-induced insulin resistance [159]. Plasma membrane-bound sn-1,2-DAG causes hepatic insulin resistance through PKCε-induced phosphorylation of IR at Thr 1160 [93]. In humans, hepatic DAG content and PKCε activity are the strongest indicators for hepatic insulin resistance in liver biopsies [160]. SFAs are also involved in ceramide biosynthesis. Increased cellular ceramide levels induce the dephosphorylation of AKT via protein phosphatase 2A, thereby negatively regulating insulin action [161]. Although previous studies showed that inhibition of ceramide synthesis reverses diet-induced insulin resistance in mice [162], there is no significant association between ceramide and hepatic insulin resistance in humans [160]. Thus, DAGs are a major lipid product of SFAs and induce insulin resistance. Unfolded protein response (UPR) or endoplasmic reticulum (ER) stress is activated in the livers of obese mice [163]. Activation of UPR using chemical inducers impairs insulin sensitivity, and attenuation of ER stress by chemical chaperones improves insulin sensitivity [163,164]. UPR-stimulated inositol requiring enzyme-1 (IRE1) activates JNK1, thus leading to phosphorylation of IRS1 at serine 307 and impairing insulin signaling [163]. In cells, lipid overload, especially SFAs, stimulates UPR signaling [165,166,167]. Therefore, SFAs also impair insulin sensitivity through the activation of UPR signaling pathways. Circulating fatty acids are sensed by the hypothalamus to maintain glucose homeostasis in a healthy condition [168]. Overnutrition or high-fat diet-induced obesity leads to brain insulin resistance and impairs systemic insulin sensitivity and glucose homeostasis in both humans and mice [169,170]. In the rodent model, brain insulin resistance occurred even after one day of high-fat diet feeding, contributing to subsequent hepatic insulin resistance [170]. Saturated fats, especially palmitic acid, impair hypothalamic insulin signaling by promoting cell membrane localization of PKC-θ [171]. Saturated fats also induce inflammation and ER stress in the hypothalamus, thereby leading to brain insulin resistance [172,173,174,175].

Long-chain polyunsaturated fatty acids (PUFAs), particularly the *n*-3 family, play a key role in regulating insulin sensitivity [176]. Several human studies have shown that omega-3 PUFAs improve insulin sensitivity in both obese non-diabetic and diabetic individuals [177,178]. Omega-3 PUFAs improve insulin sensitivity, potentially through anti-inflammation and PPAR activation. Previous studies showed that omega-3 PUFAs significantly attenuated TNF or TLR2/4 agonist-induced inflammation through GPR120 in macrophages [179,180]. Omega-3 PUFAs promote the association of GPR120 and β arrestin-2 and then induce the internalization of the GPR120/β arrestin-2 complex. The cytoplasmic β arrestin-2 interacts with TAB1 and blocks the interaction between TAB1 and TAK1, thereby inhibiting TAK1 activity, downstream IKKβ/NFκB and JNK/AP1 signaling, and improving diet-induced insulin resistance [180]. In addition, the anti-inflammatory role of omega-3 PUFAs is potentially mediated by the small lipid mediators (protectins/resolvins) that are metabolized from omega-3 PUFAs. Studies by Serhan et al. show that protectins/resolvins potentially exert an anti-inflammatory role in inflammatory diseases [181,182]. PUFAs are effective ligands for all PPAR isoforms and coordinately regulate the expression levels of genes responsible for lipid oxidation (upregulation) and lipid synthesis (downregulation), thereby improving lipid profiles and increasing insulin sensitivity [176]. C16:1n7-palmitoleate, a natural component in many foods, can be generated in adipose tissue and acts as a lipokine to increase insulin sensitivity and modulate glucose homeostasis [183]. The beneficial effect of palmitoleate is potentially mediated through GPR120 [143]. In healthy humans, ingestion of monounsaturated fatty acid (MUFA) or PUFA-enriched oil leads to higher levels of plasma GLP-1 than saturated fatty acid-enriched oil ingestion [184]. The intake of olive oil and carbohydrate meal leads to a pronounced increase in plasma GLP-1 levels, compared to the intake of butter and carbohydrate meal [185]. Ingestion of olive oil before a carbohydrate meal significantly slows gastric emptying, increases plasma GLP-1 levels, and attenuates postprandial glucose excursion [186]. An in vitro study showed that MUFAs with more than 14 carbon chain lengths significantly stimulate GLP-1 secretion in fetal rat intestinal cells [187]. Therefore, unsaturated fatty acids, including MUFAs and PUFAs, dramatically increase plasma GLP-1 levels, which may contribute to the beneficial effects of MUFAs and PUFAs on insulin sensitivity. Several cohort studies show that diabetes is more prevalent in young men than women [188,189]. Female mice are protected from high-fat diet-induced obesity and hyperglycemia, which is abolished by bilateral ovariectomy and then restored by estrogen supplementation [190]. These results suggest that estrogen plays a key role in protecting against high dietary fat-induced glucose dysregulation. Additionally, healthy young women exhibit a significantly higher GLP-1 response to intraduodenal glucose infusion than men [191]. Thus, increased GLP-1 release in females may contribute to their protection against the development of diabetes. However, whether the sex difference in the GLP-1 response is mediated by estrogen needs to be further investigated. PUFAs, particularly omega-3, improve cognitive function in both young and old individuals [192,193]. A randomized controlled trial shows that omega-3 supplementation attenuates inflammation in middle-aged and old subjects [194], which may contribute to the improvement in brain function and hypothalamic insulin sensitivity.

## 5. Protein Metabolism and Glucose Homeostasis

### 5.1. Dietary Proteins and Insulin Resistance

Dietary protein intake is important for normal growth and development. It is recommended that dietary protein accounts for 10–35% of the total diet. Numerous human studies have reported that a short-term increase in dietary protein consumption significantly decreases body weight. A high-protein diet increases satiety via the gut hormone PYY, stimulates thermogenesis [195], potentially due to the high ATP-consuming protein synthesis, and reduces subsequent energy intake, thereby leading to great weight loss [196,197]. Additionally, protein feeding induces intestinal gluconeogenesis and promotes portal glucose release, which leads to hypothalamic activation through the nerve system around the portal vein and decreases food intake [198,199]. Accordingly, short-term high dietary protein consumption stimulates insulin secretion and reduces blood glucose levels [200]. Consistently, a protein preload significantly decreases postprandial glycemia and increases plasma insulin levels in patients with type 2 diabetes, which is partially attributed to the elevation of GLP-1 and GIP levels [201,202]. A high-protein diet intervention in individuals with type 2 diabetes for 5 weeks improves glucose homeostasis [203]. However, long-term studies (over six months) in humans show that a high-protein diet is significantly associated with increased fasting glucose levels, enhanced gluconeogenesis, and impaired insulin sensitivity [204,205]. A rodent study showed that, compared to a high-fat diet, consumption of a high-fat and high-protein diet for 24 weeks significantly induces β-cell apoptosis and impairs glucose homeostasis, increasing the incidence of T2DM [206]. However, a clinical trial showed that a 12-month high-protein diet intervention has no significant effect on glucose homeostasis in subjects with type 2 diabetes [203]. Additionally, the source of dietary protein is important for the modulation of glucose homeostasis. Compared to casein, soy protein improves glucose tolerance and insulin sensitivity in rat models under high-sucrose diet feeding [207]. A cohort study showed that higher consumption of animal proteins is associated with an increased incidence of type 2 diabetes, whereas higher consumption of vegetable proteins is associated with a modestly decreased risk of type 2 diabetes [208]. The consumption of lean fish containing abundant fish protein and limited fish oil is inversely correlated with the risk of insulin resistance and T2DM [195], suggesting that fish protein potentially protects against the development of T2DM. Several studies indicate that consumption of fish protein increases insulin sensitivity and glucose tolerance, as well as improves lipid profiles [207,209,210].

### 5.2. Amino Acid and Insulin Resistance

In obese individuals, most serum amino acid levels are significantly increased, suggesting that amino acids may play a role in the regulation of insulin resistance [211]. A previous study showed that a short-term increase in plasma amino acids induces insulin resistance in skeletal muscle, decreases glycogen synthesis, and reduces whole-body glucose disposal [212,213]. Amino acid infusion stimulates phosphorylation of IRS1 at serine 312 and 636/639 via activation of the mTOR/S6 kinase 1 signaling pathway, thereby decreasing the interaction of the p85 subunit of PI3K with IRS1 and leading to insulin resistance in skeletal muscle [213,214]. In vitro studies in hepatocytes and adipocytes also show that amino acid treatment negatively modulates insulin sensitivity [215,216]. In addition, amino acids also modulate HGP to regulate glucose homeostasis. On one hand, amino acids act as substrates to contribute to gluconeogenesis. On the other hand, amino acids stimulate glucagon secretion to induce HGP indirectly [217]. These results indicate that amino acid overload may impair insulin sensitivity and glucose homeostasis by targeting the metabolic organs.

Although an amino acid mixture attenuates insulin sensitivity both in vitro and in vivo, different amino acids have distinct effects on insulin sensitivity. Branched-chain amino acids (BCAAs), including valine, isoleucine, and leucine, are critical nutrient signals in regulating body weight and muscle protein synthesis. In obese patients, plasma BCAA levels are significantly elevated [211]. Previous studies have shown that BCAAs are significantly associated with the risk of insulin resistance in humans, and BCAAs exacerbate diet-induced insulin resistance in mice [218,219,220]. Elevated BCAA levels stimulate the activity of mTOR to hamper insulin signaling via inhibitory phosphorylation of IRS1 or E3 ligase Mul1-mediated AKT2 degradation [211,218]. In addition, abnormal BCAA metabolism during obesity leads to the accumulation of toxic BCAA metabolites, thereby activating stress signaling and promoting insulin resistance [221]. Several studies have shown that muscle mitochondrial oxidative capacity is impaired in diabetic individuals [222]. Thus, dysfunctional mitochondrial BCAA catabolism potentially leads to the accumulation of BCAA metabolites in the plasma of people with diabetes, such as BCAA-derived acylcarnitines (C3 and C5), 3-hydroxyisobutyrate (3-HIB), 2-hydroxbutyric acid (2-HB), and 2-ketobutyric acid (2-KB) [221,223]. Aberrant accumulation of these metabolites causes mitochondrial dysfunction, lipotoxicity, and insulin resistance [223,224,225,226]. On the other hand, increased BCAA and its metabolites inhibit the activity of the pyruvate dehydrogenase complex (PDH) in the liver and heart, potentially through increased BCAA-derived acetyl-CoA, leading to a significant decrease in glucose uptake and oxidation [221,227]. Thus, BCAA may regulate glucose homeostasis through modulating the activity of PDH. In contrast to BCAAs, plasma glycine levels are significantly reduced in patients with obesity or diabetes [211,228]. Several studies indicate that a low plasma glycine level is a predictor for insulin resistance and T2DM [229,230,231]. Glycine treatment decreases hepatic oxidative stress marker levels, potentially via increasing glutathione concentrations, thereby improving insulin sensitivity [232]. Arginine is a precursor for nitric oxide. A previous study showed that long-term arginine administration improved insulin resistance in individuals with T2DM [233], suggesting that arginine has a beneficial effect on glucose homeostasis. Indeed, our unpublished data showed that arginine attenuated glucagon-induced HGP through FoxO1. However, the underlying mechanism by which arginine regulates glucose homeostasis needs to be further investigated. Considering the different effects of amino acids on insulin sensitivity, the composition of amino acids in the body plays an important role in the regulation of insulin resistance. This is supported by the evidence that the beneficial effect of cod protein on insulin resistance is recapitulated by an amino acid mixture identified in cod protein-treated rat plasma [210].

## 6. Precision Nutrition and Nutrigenomics

Precision nutrition is becoming an important aspect of metabolic syndrome management. The purpose of precision nutrition is to design a unique nutritional recommendation for each individual according to the combination of an individual’s genetics, metabolome, microbiota, and lifestyle factors, thereby preventing metabolic diseases [234]. Based on the International Society of Nutrigenetics/Nutrigenomics (ISNN), precision nutrition should be considered to develop at the following three levels: (1) conventional nutrition based on a general guideline for different populations categorized by age, gender, and other social determinants; (2) individualized nutrition developed by a deep and refined phenotype, including anthropometry, metabolic analysis, physical activity, and others; (3) phenotype-guided nutrition based on the effect of genetic variants or gene expression and their impact on individual responses to specific nutrients [235]. Nutrigenomics aims to investigate the crosstalk among nutrients, diet, and gene expression [235]. The advances in “Omics” technologies, including genomics, transcriptomics, proteomics, metabolomics, metagenomics, and epigenomics, offer a comprehensive method to analyze how specific nutrients affect gene expression and subsequent metabolic responses. Nutrigenetics has provided a better understanding of nutrient or diet effects on metabolic diseases via the discovery of disease-associated genetic variants [235]. The implementation of these disease-associated genetic variants helps to better understand the heterogeneity in nutrient response among different individuals and provides support to develop more precise dietary recommendations in clinical practice. Several studies have shown that macronutrient intake affects the association of genetic markers with metabolic symptoms. Martinez’s group evaluated the interaction between diet and genetics using a genetic risk score (GRS) calculated based on 16 metabolic syndrome-associated SNPs. They found that macronutrient intake modified the GRS association with metabolic traits [236]. Other studies also showed that total protein intake and sugar-sweetened beverages modulated the genetic association with obesity [237,238]. Individual genetic information is invaluable for tailoring the most suitable dietary interventions for the prevention or treatment of metabolic diseases. Currently, genetic tests have been used to customize individual diets based on their response to specific nutrients, such as caffeine metabolism-associated SNPs and saturated fat-sensitive APOA2 polymorphism [239,240].

Metabolomics provides a more accurate understanding of the metabolism of nutrients and the impact of nutrients on an individual’s health status. Identifying food-derived biomarkers and standardizing the metabolite reference value are important for the application of metabolomics to precision nutrition [234]. Metabolomics is a pillar of deep phenotyping. Metabolic phenotyping based on metabolomics will identify individuals with different responses to interventions, which will help tailor dietary recommendations to the individual [241]. The gut microbiota is affected by antibiotic use and dietary intake, which helps to customize personalized medicine and nutrition. The composition of the microbiota is unique to each individual and represents that individual’s metabolic status. A previous study showed that individuals with low bacterial richness are characterized by significant adiposity and insulin resistance [242]. This result suggests that the gut microbiota is a potential target for the treatment of obesity and associated metabolic diseases. The composition and diversity of gut microbiota can be modulated by macronutrient intake, the host genetic makeup, and the interaction between diet and host genetics [243]. Thus, gut microbiota profiling is an important aspect of nutritional intervention. It is promising to perform an integrative analysis of the gut microbiota and metabolomics dataset to evaluate adherence to healthier dietary patterns [244]. Although it is a huge challenge to perform and interpret the integrative multi-omics dataset analysis, there is no doubt that the information we get from multi-omics approaches will improve our understanding of diet-gene-disease interaction and promote the application of precision nutrition in clinical practice.

## 7. Conclusions

**Figure 2:**
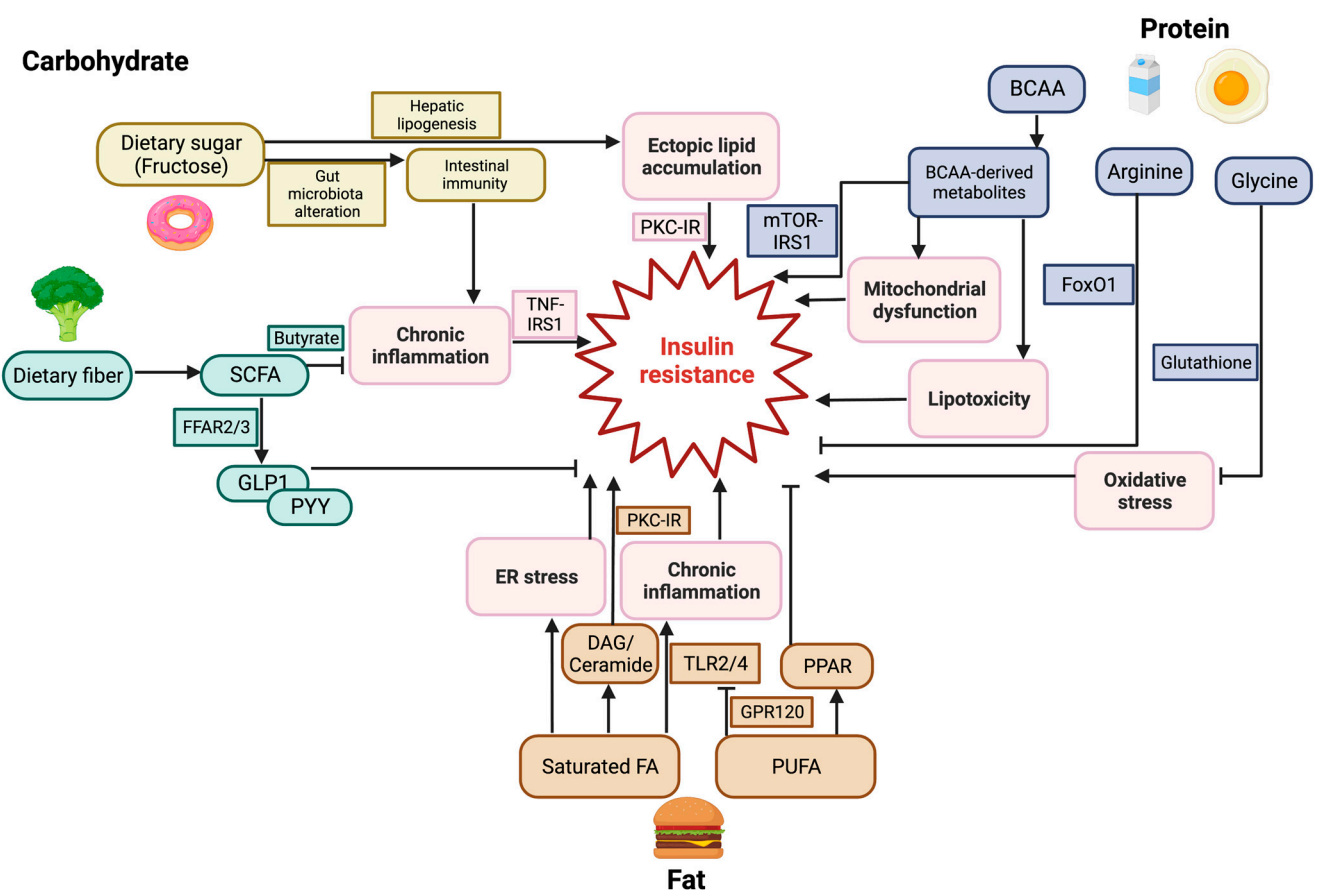
Macronutrients regulate insulin resistance and glucose homeostasis through distinct molecular mechanisms. Fructose leads to insulin resistance by increasing hepatic lipogenesis and impairing gut immunity. Dietary fiber improves insulin sensitivity through gut microbiome-derived SCFAs. Increased dietary fat intake elevates free fatty acid levels, especially unsaturated fatty acids, thereby attenuating insulin sensitivity by inducing pro-inflammatory activity and activating DAG-PKC signaling. Increased BCAA levels induce insulin resistance through activation of mTOR-IRS signaling and BCAA metabolite-induced oxidative stress. Glycine improves insulin sensitivity potentially through the generation of glutathione, and arginine contributes to insulin sensitivity by inhibiting FoxO1. BCAA, branched-chain amino acid; DAG, diglyceride; ER stress, endoplasmic reticulum stress; FA, fatty acid; FFAR, free fatty acid receptor; FoxO1, forkhead/winged helix transcription factor O-class member 1; GPR120, G-protein coupled receptor 120; GLP-1, glucagon-like peptide-1; IR, insulin receptor; IRS, insulin receptor substrate; mTOR, mammalian target of rapamycin; PYY, peptide tyrosine tyrosine; PKC, protein kinase C; PPAR, peroxisome proliferator-activated receptor; PUFA, polyunsaturated fatty acid; SCFA, short-chain fatty acid; TLR, toll-like receptor; TNF, tumor necrosis factor. →: Activation; 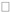: Inhibition.

T2DM is marked by systemic insulin resistance and β-cell failure-induced insulin deficiency. Overnutrition promotes insulin resistance at multiple levels, including insulin receptor desensitization at the cell surface, inhibition of IRS function through degradation, suppression of PI3K activity, failure to control FoxO1-induced transcriptional changes, and reduced insulin clearance from the bloodstream. Dietary carbohydrate intake induces insulin secretion, and overconsumption of carbohydrates leads to insulin resistance. Fructose is a major driver for the development of insulin resistance through increasing hepatic lipogenesis and impairing gut immunity. Dietary fiber improves insulin sensitivity through gut microbiome-derived SCFAs. Increased dietary fat intake elevates free fatty acid levels, especially unsaturated fatty acids, thereby attenuating insulin sensitivity by inducing pro-inflammatory activity and activating DAG-PKC signaling. PUFA improves insulin resistance through suppression of TLR2/4 signaling and activation of PPAR signaling. The composition of amino acids after dietary protein intake plays a pivotal role in insulin sensitivity and glucose homeostasis. Particularly, increased BCAA levels induce insulin resistance through activation of mTOR-IRS signaling and BCAA metabolite-induced oxidative stress (Figure 2). Short-term protein feeding improves glucose homeostasis, which may be attributed to the increased release of gut hormones, including GLP-1 and GIP. Precision nutrition aims to design a unique nutritional recommendation for each individual according to the combination of an individual’s genetics, metabolome, microbiota, and lifestyle factors, thereby preventing metabolic diseases. Understanding the molecular mechanisms of diet-gene interactions will help us apply precision nutrition to clinical practice by integrating multi-omics approaches.
